# Improving the management of acute asthma in children through an integrated care pathway: an implementation study protocol

**DOI:** 10.3389/fped.2025.1646499

**Published:** 2025-08-19

**Authors:** Marta Montejo, Natalia Paniagua, Alvaro Sanchez, Mikel Rueda-Etxebarria, Jose I. Pijoan, Carlos Saiz Hernando, Vanesa Martin, Miguel Angel Vázquez Ronco, Mikel Santiago Burruchaga, Diego García Urabayen, Garbiñe Pérez Llarena, Susana Castelo, Javier Benito

**Affiliations:** ^1^ San Vicente Health Centre, Barakaldo-Sestao Integrated Health Organization, Basque Health Service-Osakidetza, Barakaldo, Spain; ^2^ Paediatric Emergency Department, Ezkerraldea-Enkarterri-Cruces Integrated Health Organization, Cruces University Hospital, Basque Health Service-Osakidetza, Barakaldo, Spain; ^3^ Primary Care Research Unit of Bizkaia, Deputy Directorate of Healthcare Assistance, Biobizkaia Health Research Institute, Basque Health Service-Osakidetza, Barakaldo, Spain; ^4^ Network for Research on Chronicity, Primary Care, and Health Promotion (RICCAPS), Barakaldo, Spain; ^5^ Biobizkaia Health Research Institute, Osakidetza-Basque Health Service, Barakaldo, Spain; ^6^ Clinical Epidemiology Unit, Cruces University Hospital, Basque Health Service-Osakidetza, Biobizkaia Health Research Institute, Barakaldo, Spain; ^7^ CIBER of Epidemiology and Public Health (CIBERESP), Madrid, Spain; ^8^ Department of Medical Documentation, Cruces University Hospital, Basque Health Service-Osakidetza, Biobizkaia Health Research Institute, Barakaldo, Spain; ^9^ Quality and Innovation Unit, Barakaldo-Sestao Integrated Health Organization, Basque Health Service-Osakidetza, Barakaldo, Bizkaia, Spain; ^10^ Paediatric Hospitalization, Ezkerraldea-Enkarterri-Cruces Integrated Health Organization, Cruces University Hospital, Basque Health Service-Osakidetza, Barakaldo, Spain; ^11^ Paediatric Pulmonology and Cystic Fibrosis Unit, Ezkerraldea-Enkarterri-Cruces Integrated Health Organization, Cruces University Hospital, Basque Health Service-Osakidetza, Barakaldo, Spain; ^12^ Paediatric Intensive Care Unit, Ezkerraldea-Enkarterri-Cruces Integrated Health Organization, Cruces University Hospital, Basque Health Service-Osakidetza, Barakaldo, Spain; ^13^ Innovation Unit, Ezkerraldea-Enkarterri-Cruces Integrated Health Organization, Basque Health Service-Osakidetza, Barakaldo, Spain

**Keywords:** asthma attack, asthma exacerbation, integrated care pathway (ICP), implementation, pediatrics, primary care, emergency departement, effectiveness

## Abstract

**Introduction:**

This study aims to evaluate the effectiveness and feasibility of the implementation of an Asthma Integrated Care Pathway (AICP) to improve care and reduce variability according to recommended clinical practice guidelines, for children presenting with acute asthma episodes in paediatric primary care, and hospital services including emergency departments.

**Methods and analysis:**

A cluster quasi-experimental implementation trial with a matched control group will be launched in a regional healthcare service. All the paediatric healthcare professionals providing care in two health districts (HDs) will receive interventions over two 12-month periods during which components of the implementation strategy to favour adoption of the AICP will be deployed cumulatively. A selected set of professionals from the same levels of care in the other HDs (*N* = 11) will serve as the comparison group. The target population of the AICP is children between 2 and 14 years old presenting with an acute asthma episode during the study period. A mixed methods evaluation guided by the RE-AIM framework will assess the effectiveness of the AICP after 12, 24, and 36 months in a set of pre-specified care and implementation outcomes at the professional level. The perceived feasibility of the AICP and its implementation from the perspective of physicians and the experience and satisfaction of patients concerning the clinical care received will be assessed through discussion groups.

**Discussion:**

This study performed in real-world settings will contribute in extending knowledge in asthma care pathways beyond emergency settings into primary care and across the healthcare continuum. In addition, its findings aim to guide health systems in reducing variability in care, increasing guideline adherence, and ultimately improving paediatric asthma outcomes across the system.

**Clinical trial registration:**

ClinicalTrials.gov, identifier [NCT06437444].

## 1 Introduction

Asthma is the most common chronic disease in childhood, with a prevalence of up to 20%, depending on the geographical area (1–3). Poor disease control can lead to substandard quality of life, missed days at school, missed workdays for caregivers, visits to paediatric emergency departments (PEDs), and hospital admissions. Around 20% of children with asthma experience asthma attacks (AAs) (also called asthma exacerbations) that require medical attention, both in primary care (PC) and PEDs (4–6). Up to a 15% of patients require hospital admission (6, 7), and it is estimated that they account for around 5% of PED visits. AAs account for more than 80% of the direct costs associated with asthma (7–10). Numerous clinical practice guidelines (CPGs) have been developed with recommendations to mitigate the impact of AAs (11–14). However, difficulties in adhering to these recommendations have been reported in both PEDs and PC services (15–18).

Integrated care pathways (ICPs) aim to translate CPG recommendations into clinical processes of care within the unique culture and environment of a healthcare institution, to reduce variation, improve quality of care, and optimize outcomes for specific patient groups (19–21). In line with this, it appears that asthma care pathways have the potential to improve the management of asthma in children, as they seem to have positive results in achieving better adherence to recommended care, resulting in more efficient and homogeneous care. More specifically, it has been demonstrated that paediatric asthma care pathways implemented in PED settings could help to improve the quality of care (22) by increasing the use and timely administration of recommended medications (bronchodilators and systemic corticosteroids) (23–25), decreasing length of PED stays (25–28), decreasing risk of hospital admission (23), and increasing training in asthma management (24). Nonetheless, all asthma care pathways reported have been implemented in only one specific context, mainly in PEDs and hospital care. This limited scope, together with the recent experience of our research group with the successful implementation and scaling up of a care pathway for the management of acute bronchiolitis (29), has led us to develop a new integrated care pathway for the management of AAs at all levels of care. To this end, when designing our implementation strategy, we have also taken into account the potential barriers detected in previously implemented asthma pathways both in PC (30) and hospital care [27], such as lack of awareness of the care pathway, lack of integration of the pathway into clinic flow, or difficulty in modifying EHR or in obtaining consensus on practice changes.

The general objective of this study protocol is to evaluate the effectiveness and feasibility of the implementation of the Asthma Integrated Care Pathway (AICP) to improve the care provided to children presenting acute asthma episodes and to reduce the variability among PC and PED professionals and care settings in accordance with clinical practice guideline recommendations (11–14).

The specific objectives will be:

### 1.1 Effectiveness objectives

To evaluate the effectiveness of the implementation of the AICP on: (a) increasing the rate of administration of bronchodilators using a metered-dose inhaler (MDI) with a spacer chamber in children diagnosed with a mild-to-moderate AAs in PC, PEDs, and hospital wards; (b) increasing the Pulmonary Score documentation rate at all levels of care; (c) increasing the rate of recording asthma symptoms using the Pediatric Asthma Control Tool (PACT) (31) in PC and PEDs; and (d) increasing the rate of initiation of background treatment in children with persistent asthma symptoms, in PC and PEDs.

### 1.2 Implementation objectives

(a). To assess the reach of the AICP among the population eligible to receive it 12 months after its implementation; determine the extent to which the exposed population is representative of the target population; and ascertain the perception and experience of exposed users (family members) regarding the quality of care received for the management of AAs and the response to their concerns and needs (REACH)
(b). To assess the degree of adoption of the AICP by professionals/centres 12 months after its implementation; and determine the degree of representativeness of adopting professionals/centres and the main barriers or difficulties for adoption (ADOPTION)
(c). To assess the degree of implementation of the AICP components, their fidelity to the protocols, and any adaptations made; describe the set of actions or strategies necessary for its adequate implementation and the level of exposure of the implementing agents to them; and assess the feasibility and acceptability perceived by professionals regarding the implementation of the pathway, with special emphasis on the identification of barriers, facilitators, and strategies needed to inform future system-wide scaling up (IMPLEMENTATION)
(d). To assess the sustainability of AICP implementation in the long term (24 months), resources, and adaptations required (MAINTENANCE).

## 2 Methods and analysis

### 2.1 Design and timeline of the study

**Figure 1:**
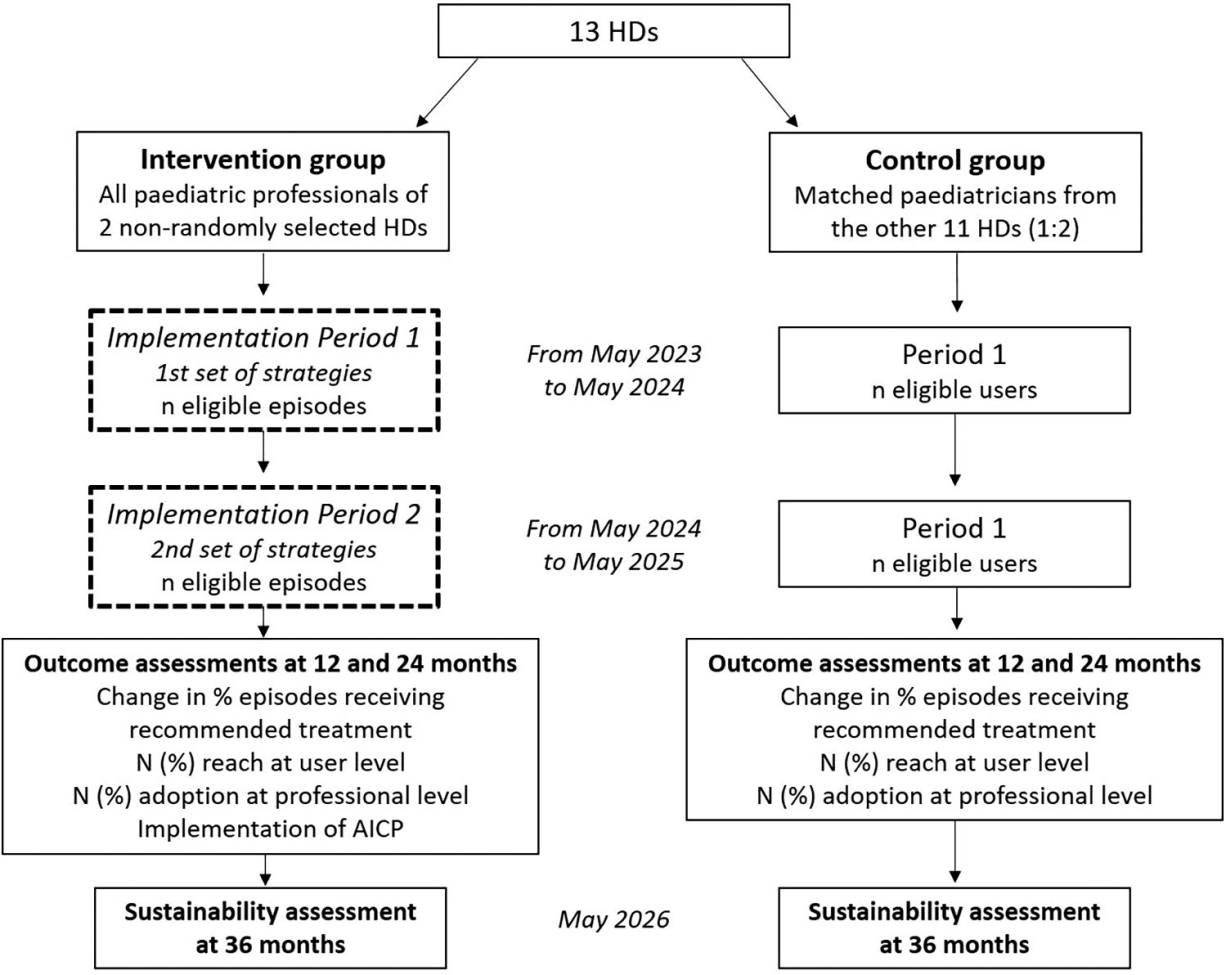
Study design diagram. HD, health district; AICP, asthma integrated care pathway.

A cluster quasi-experimental implementation trial with matched control group evaluated with mixed methods will be conducted in paediatric services of Basque Health Service, Osakidetza. The present study do not imply prospective recruitment of patients as it consists in a study performed under real world circumstances of clinical practice regarding acute asthma management by professionals of participating paediatric services. A two 12-month implementation periods will take place between May 2023 and May 2025, during which components of the implementation strategy to favour adoption of the AICP will be cumulatively deployed. This will involve, first, a set of strategies facilitating initial uptake (from 7 May 2023 to 7 May 2024); and second, a set of strategies related to broad dissemination and communication among professionals and healthcare centres involved (from 7 May 2024 to 7 May 2025) (see Supplementary File S1 for more detailed specification). The quantitative evaluation to assess the results of the implementation of the AICP at professional and patient levels will be carried out considering the year prior to the first implementation period as the baseline measurement, and three post-intervention measurements at 12, 24 and 36 months from initial implementation of the pathway until May 2026. A flow diagram with further description of the study phases is presented in Figure 1. Final quantitative results of study are expected in June 2026. The qualitative evaluation will be carried out upon the completion of the second implementation period through a structured process with discussion groups. These groups will be focused, on the one hand, on the identification of the main barriers and facilitators for the provision of recommended clinical practice by the healthcare professionals, and on the other, on patients’ (and their relatives’) experience and satisfaction with the clinical care received.

### 2.2 Setting

The study will be carried out mainly in 2 HDs, those of Ezkerraldea-Enkarterri-Cruces and Barakaldo-Sestao, out of the 13 districts that make up our regional health service, which provides comprehensive universal health coverage, free at the point of delivery. These HDs each have a PC area and share a reference hospital. Therefore, the care pathway will be implemented in two PC areas and the PED, hospital wards, paediatric intensive care unit (PICU), and paediatric pulmonology department of their reference hospital. In the two HDs as a whole, the PC consultations deal with around 2,000 episodes of AAs per year. The reference PED deals with around 3,000 episodes of AAs per year (5% of all episodes). Selected professionals from the same levels of care in the other 11 HDs will be used as the comparison group.

### 2.3 Participants

Eligibility criteria for the study will be as follows:

#### 2.3.1 Professionals

All healthcare professionals providing paediatric care in Osakidetza. This includes all paediatricians and nurses caring for paediatric AAs in PC, PEDs, hospital wards, and PICUs.

#### 2.3.2 Patients

All acute asthma episodes attended by any participating paediatric healthcare professionals during the study field period. An acute AA is defined as an acute episode of wheezing in a child between 2 and 14 years with a previous diagnosis of asthma or a history of wheezing; or a first episode in a child between 2 and 14 years with a personal/family history of atopy and/or an objective response to bronchodilators as assessed by the Pulmonary Score.

### 2.4 Allocation to intervention and comparison groups

The paediatric professionals working in PC, the PED, hospital wards, the PICU, and the paediatric pulmonology department of Ezkerraldea-Enkarterri-Cruces and Barakaldo-Sestao HDs will constitute the intervention group and will receive the implementation strategy for the AICP. The comparison group will be made up of paediatric professionals from the other 11 HDs who will not receive any interventions. To increase comparability and reduce potential biases, two professionals from the comparison HDs will be selected for each paediatrician in the intervention group by matching in terms of paediatrician-related characteristics (e.g., baseline outcome rates, age, sex, etc.) and the characteristics of the patient population assigned to the paediatrician (size, mean age, male/female ratio, mean socioeconomic status, mean comorbidity level, etc.). This matching strategy used in the study aims to address the two main problems related to comparisons with non-randomized groups: (a) historical bias, in which events unrelated to the intervention (also referred to as secular trends) occur before or during the intervention period and have an effect on the outcome (either positive or negative) that is unrelated to the intervention; and (b) differences between intervention and control professionals, as non-equivalent control groups differ from those in the intervention in a number of significant ways that impact the outcome of interest and may bias the results (selection bias).

### 2.5 Clinical intervention

The AICP is a structured multidisciplinary care plan that details the essential evidence-based steps in the care of patients with mild-to-moderate AAs and the coordinated practice of the agents involved as dictated by the evidence. In addition, the pathway determines the target population (inclusion/exclusion criteria); the sequence of common best practices during the patient's navigation through the system, according to the best available evidence in terms of: diagnostic methods and criteria, as well as their coding and recording; the provision of care and recommended clinical practices (actions, responsibilities, etc.); the educational activities for the patient and their relatives; the management of the patient and the patient's care; complex situations, management of difficulties and referral criteria; due attention to transitions between care levels and communication between professionals; and the outcomes to be pursued, evaluated and monitored.

In essence, the four core components of evidence-based clinical practice related to acute asthma as stated in the AICP (see Supplementary File S2) are:

• Assessment and recording of the Pulmonary Score;
• Assessment and recording of asthma symptoms using the PACT;
• Administration of background treatment in cases of persistent asthma symptoms;
• Administration of bronchodilators using an MDI with a spacer chamber in children diagnosed with a mild-to-moderate asthma attack.

The AICP was built on the model followed in the design of the Integrated Acute Bronchiolitis Care Pathway (29). It was developed based on the consensus of professionals involved in caring for children with asthma at different levels of care and the advice of experts in quality and medical documentation. Work started in 2020 and after an external evaluation process, its first version was approved in April 2021 (under editorial review).

### 2.6 Implementation strategy

The implementation strategy to promote the adoption of the AICP is based on the one already proven effective in scaling up the Acute Bronchiolitis Pathway (29). It was also developed based on the consensus of professionals involved in caring for children with asthma at different levels of care as well as the advice of experts in quality improvement and implementation research. A Logic Model outlining how the AICP and the accompanying implementation strategy is expected to work can be found in the Supplementary File S3.

The strategy will be deployed in two implementation periods and consists of the following active components (see Supplementary File S1):

(1) First implementation period:
  • Initial dissemination of the AICP document and support materials through several web seminars (using the ZOOM platform) for professionals within the organizations involved
  • Integration of information and communication tools into electronic health records (EHRs) to facilitate the recording and the standardized implementation of recommended practice actions, including access to educational material to be provided to patients and families
  • Delivery of training courses for paediatricians and paediatric nurses
  • Distribution of monthly audit/feedback reports with data on changes in the AICP indicators at their health centre level and those obtained in other centres in the participating health areas
  • Circulation of newsletter notifications with AICP-related messages.
(2) Second implementation period (in addition to the previous strategies):
  • Multi-channel dissemination of the AICP to professionals through various communication and training actions: General presentations to professionals within organizations involved through several web seminars (ZOOM sessions); dissemination and review of the active components and innovative aspects of the AICP via videos available through the intranet and online; weekly training “pills” through corporate social networks on epidemiological data and data regarding the health, economic and social impact of the AICP; key messages on current treatment recommendations based on the latest CPGs and the recommended protocol; and reminder posters distributed to all paediatric services, the PED and hospital outpatient clinics.
  • Dissemination to healthcare users: reminder posters distributed to all paediatric services, the PED, and hospital outpatient clinics with QR codes for accessing educational content (e.g., videos).

### 2.7 Outcome measures

The impact evaluation of the AICP implementation will be conducted using the Reach, Effectiveness, Adoption, Implementation, and Maintenance (RE-AIM) framework, an evaluation framework developed by Glasgow et al. (32) to extend the assessment of interventions beyond effectiveness or efficacy to multiple criteria that can better identify the public health impact of health promotion programmes, initiatives or interventions.

### 2.8 Evaluating the effectiveness of the AICP

The main effectiveness outcome will be the change from baseline to 12 and 24 months in the rates of mild-to-moderate AA episodes seen in PC and the PED in which patients are administered bronchodilators using a MDI with a spacer chamber. We will assess the overall effect of the AICP by testing the interaction between intervention and time of measurement. Should this intervention-by-time interaction be significant (with *p* < 0.05 as the threshold for significance), planned comparisons will be performed to determine whether changes in the AICP group between baseline and each of the follow-up points are significantly different from those observed in the control group. Secondary effectiveness outcomes will be the change from baseline to 12 and 24 months after the implementation of the AICP in: (a) rate of recording of Pulmonary Score at all levels of care; (b) rate of recording of asthma symptoms using the PACT at all levels of care; and lastly, (c) the change in rate of persistent asthma symptoms in children who initiate a background treatment at all levels of care.

### 2.9 Evaluating the implementation of the AICP

#### 2.9.1 REACH

Absolute number and percentage of episodes of patients between 2 and 14 years presenting with an acute asthma episode during the study field period seen by any participating paediatric healthcare professionals exposed to the clinical practice established by the AICP for the management of AAs 12 and 24 months after its implementation, and their representativeness (characteristics of the participants compared to those of the target population). We consider that an episode has been exposed to the AICP if the patient has at least one of the recommended practices in an asthma episode registered: (a) assessment and recording of the Pulmonary Score; (b) assessment and recording of asthma symptoms using the PACT, (c) administration of background treatment in cases of persistent asthma symptoms, and/or (d) administration of bronchodilators using an MDI with a spacer chamber in children diagnosed with a mild-to-moderate asthma attack.

#### 2.9.2 ADOPTION

Absolute number and percentage of paediatric healthcare professionals adopting the AICP for the management of AAs 12 and 24 months after implementation, and their representativeness (characteristics of professionals/centres adopting the intervention/programme compared to those of professionals/centres potentially eligible to participate in the intervention/programme) (characteristics of adopters/non-adopters). We consider that a professional has adopted the pathway if he/she has registered at least one of the recommended practices in an asthma episode (assessment and recording of the Pulmonary Score; assessment and recording of asthma symptoms using the PACT; administration of background treatment in cases of persistent asthma symptoms; and/or administration of bronchodilators using an MDI with a spacer chamber in children diagnosed with a mild-to-moderate asthma attack) in at least 50% of the episodes attended. This adoption threshold, as a desirable goal for improvement, was proposed by the clinical committee within the research team and agreed with a representation of professionals from different levels of care after having considered baseline levels.

#### 2.9.3 IMPLEMENTATION

The degree of fidelity with which the professionals/centres have implemented the AICP and its components for the management of AAs compared to what was planned will be evaluated. For this purpose, a complete record and subsequent description will be made of the implementation process, and any adaptations made to the AICP or its components. In addition, the fidelity of the implementation strategies designed to facilitate the adoption of the AICP by professionals/centres will be evaluated. To this end, we will provide a complete record and subsequent description of each strategy's execution process and documentation of adaptations made. Further, to assess dose, quality of delivery, professionals’ responsiveness, and programme differentiation (33), the exposure of professionals/centres/centres to the strategies will be measured by a questionnaire developed ad-hoc for this project.

In addition, a representative sample of professionals from the different levels of care involved (PC, PEDs, hospital wards, PICU, and paediatric pulmonology department) with high and low adoption and implementation rates of the good practice criteria established by the AICP (administration of bronchodilator with a spacer chamber; assessment and recording of the Pulmonary Score; and assessment and recording of persistent symptoms - asthma severity) will participate in a structured process using a qualitative methodology (discussion groups) focused on identifying the main barriers and facilitators for the provision of recommended clinical practice. A purposive sample of paediatricians will be recruited stratified by level of care seeking to ensure that all views are represented. All discussion groups will count with at least 6 professionals organized by level of care. Since one of the objectives is for the professionals to express their group perception of the implementation of the AICP at their level of care, we seek to achieve intra-group homogeneity and therefore the possibility of mixing people from different levels of care in the same group is not contemplated. Sampling will continue until saturation is reached, defined as two consecutive discussion groups in which no new additional information is gathered.

The groups will be led by two researchers with experience in qualitative research, as well as knowledge of the clinical field and the project. The discussion groups will be recorded, with prior consent, and transcribed verbatim. The discussion group scripts will explore in detail the determinants of prescribing practice, with questions formulated to explore each of the 14 domains of the Theoretical Domains Framework (TDF) (34, 35). The script will be developed by researchers with expertise in behaviour change and implementation research. The discussion group script and operational procedure will be piloted and refined before the fieldwork. The transcribed texts will be independently reviewed and analysed by two researchers using qualitative techniques derived from discourse analysis with a deductive perspective. The script will be developed by researchers with expertise in behaviour change and implementation research. The discussion group script and the operational procedure will be piloted and refined before the fieldwork. The transcripts will be independently reviewed and analysed by two researchers using qualitative techniques derived from discourse analysis with a deductive perspective. They will used NVivo 15.0 software (QSR International) to manage the data and facilitate analysis. The data will be analysed using an iterative process in which two researchers will independently review the group transcripts and coding using thematic content analysis related to the factors included in the TDF. First, the thematic categories will be identified using the coding guide related to TDF dimensions developed for the study (see Supplementary File S4). Second, the relevance of each TDF construct in the discourse of the discussion group of professionals from each healthcare field will be analysed. Specifically, a score (ranging from −2 to +2) will be assigned to each construct to highlight its importance in the professionals’ discourse (whether it is a topic that appears or does not appear) and the meaning of the association (i.e., whether it is mentioned in positive terms or as a facilitator, or in negative terms as a barrier).

Themes emerging outside the TDF domains will be object of an inductive analysis based on grounded theory (36). To do so, first, recurring words, concepts and themes emerging from participants’ discourse, will be identified and coded within the data. Next, similar codes will be grouped together to form broader themes or categories. Finally, the identified themes will be interpreted, and potential relationships between them will be explored to develop a deeper understanding of the phenomenon under study and to favour the development of a theory on the functioning of the implementation strategy. Any discrepancies in coding or scoring among researchers thorough the present qualitative analysis will be discussed until final consensus is reached.

Furthermore, discussion groups will be organized to explore the perception and experience of the exposed users (family members) regarding the quality of care received for the management of AAs and the response to their concerns and needs. The procedure will be similar to that used with professionals. The sample will include at least 20 relatives or legal guardians of patients treated for episodes of mild-to-moderate AAs at the 5 levels of care involved (at least 4 per level). Specifically, two or three discussion groups will be held with at least eight participants in each. In order to gather insights into the experiences and perspectives of several demographic characteristics, factors such as age, gender or socioeconomic status will be considered during recruitment. Moreover, to assure experiential diversity, at least half of the participants will be relatives of patients who have received care in accordance with AICP, and the other half, relatives of patients in whom such recommended practice did not take place. In this way, within each group, heterogeneity among patients will be examined seeking to better understand their satisfaction and experience with the clinical performance and identify the factors felt to be important by users.

Finally, qualitative analyses will be shared with all participants in order to ensure and validate that they accurately reflect their experiences and perspectives.

#### 2.9.4 Maintenance

The sustainability of the AICP implementation will be assessed 36 months after its initial implementation. This will include a complete record and subsequent description of the implementation process (indicators of Reach, Adoption, Implementation), whether the AICP continues to be implemented (institutionalized or not), the resources required for its maintenance, and any adaptations made to the pathway or the implementation strategies. In addition, the change in primary and secondary effectiveness variables will be measured as an indicator of improvement in the management of AAs from 24 to 36 months after the start of the AICP implementation (long-term effectiveness).

### 2.10 Data management plan

This study will be carried out in accordance with international standards for the conduct of epidemiological studies, included in the International Guidelines for Ethical Review of Epidemiological Studies (37). In order to learn about the management of AAs, data will be collected from the health centres and the PED and inpatient care-paediatric pulmonology reference centres of the two HDs. This is a prospective intervention study, where the data will be collected from the EHR, using the data exploitation program of our regional health service (OSABIDE Global). The Primary Care Research Unit of Bizkaia is formally authorized by the Healthcare Directorate of Osakidetza to extract and use data from the EHR for research purposes. A diagnostic search will be carried out for the term asthma with AAs (International Classification of Diseases code J45.901). Data will also be gathered on the number of prescriptions given for inhaled bronchodilators, or inhaled and systemic corticosteroids, the recording of severity using the Pulmonary Score, and the use of a spacer chamber in mild-to-moderate attacks. In the case of prescriptions, only those made using the Basque Health Service prescription platform and associated with AA episodes will be recorded. As this data collection and recording will be performed without naming and without any participation of the patients as it is mainly routine clinical practice data, need for consent was waived by the ethics committee. Conversely, participants in the structured focus group meetings (both professionals and patients’ relatives/caregivers) will be informed about the study and their written informed consent will be obtained concerning the information directly collected from them (see Supplementary Files S5, S6).

All the information regarding the study subjects, either that extracted from EHRs for this research or collected from the participants of focus groups, will be protected and treated confidentially for all purposes, in accordance with the provisions of the Spanish Organic Law 3/2018, of 5 December, on Personal Data Protection and the guarantee of digital rights (LOPD-GDD) and the provisions of Regulation (EU) 2016/679 of the European Parliament and of the Council of 27 April 2016, on the protection of natural persons with regard to the processing of personal data and on the free movement of such data (General Data Protection Regulation). Specifically, all data will be anonymously documented, de-identified, and linked to unique codes that are meaningless outside the context of the system. The resulting database will be exported to a formatted plain text file that will be compressed and encrypted using a secure algorithm, and then, processed and included in a robust and secure database server.

### 2.11 Analysis

Frequencies and proportions along with the corresponding 95% confidence intervals (CIs) will be used to describe the rate of administration of bronchodilators using an MDI with a spacer chamber in paediatric asthma patients at all measurement points. The primary effectiveness outcome will be the changes in this bronchodilator use rate in children diagnosed with mild-to-moderate AAs in primary care, the PED, and hospital wards. To test the overall effect of the AICP, we will compare changes in outcome variables between the two groups over the three follow-up measurements adjusted for baseline values. Given the binary nature of most response variables (prescription rates or performing certain clinical practices/actions), the underlying regression model will be a logistic regression model, and hence, odds ratios or risk ratios will be calculated as estimators of the effect of the intervention. Multivariate mixed logistic models will be used to take into account the hierarchical structure of the data, with patients nested in paediatricians and/or health centres in each of the measurement periods (SAS PROC GLIMMIX ver. 9.4, SAS Institute, Cary, NC, USA, 2023). Separate models will be fitted for data from PC centres and EDs due to differences in the hierarchical structure of the data. In both PC and ED-related models, the AICP intervention, the time of measurement and intervention-by-time interaction will be included as fixed effects. Paediatricians and centres will be included as random effects in the intercept and the slope of the different repeated measurements in PC models, while a structure of patients nested in hospitals will be fitted for ED data, as patient care at EDs cannot be assigned to any specific physician. In all mixed models fitted, restricted maximum likelihood ratio tests and the Akaike and Bayesian information criteria will be used to estimate the modelled parameters (fixed and random), and to determine the best covariance structure for our data, respectively. Backward selection of effects with *p* < 0.05 as a threshold for inclusion and Laplace approximation will be used to identify parsimonious final multivariate mixed logistic models.

Additional propensity score analysis aimed to reduce potential bias related to the non-randomly generated comparison group will be used. This statistical procedure involves forming matched sets of subjects by comparison group who share a similar value of the propensity score (38). In the present study, this analysis will be carried out using the logit function of the probability of belonging to the intervention group, determined by a series of prognostic factors related to paediatric professionals (e.g., baseline rate of episodes in which bronchodilators are administered using a MDI with a spacer chamber) or to the characteristics of the episodes attended by the professionals (e.g., average age, etc.). A 2:1 matching ratio (two matched professionals from the comparison group for each intervention group's professional) and no replacement strategy through nearest neighbour matching with a calliper width that ensures the planned matching ratio (e.g., 0.2) will be used (38). In order to assess balance among matched subjects, standardized mean differences will be used. Finally, to estimate and compare results PROC GLIMMIX (random int/subject = match_id) will be used as a method to match the pairs and appropriately calculate the variance.

Considering an average of 1,100 AA episodes per year attended in PC within the participating two HD, and assuming a 10% rate of episodes in which bronchodilators administered using a MDI with a spacer chamber in the reference group, using a two-tailed chi-square test for two independent samples with a significance level of 5%, and a variance inflation factor due to the clustering of 20 episodes per professional, the present study will have a 80% power to detect as statistically significant a 50% increase in the rate of episodes with bronchodilators administered using a MDI with a spacer chamber (absolute proportion 15%) occurred in the intervention group.

## 3 Discussion

The present study aims to improve the care of children with asthmatic crisis and to reduce the existing variability among professionals and the various health care services. More specifically, the project seeks to establish a homogeneous evidence-based care in episodes of asthmatic crises in children at all levels of care through the implementation of the AICP.

A strength of this non-randomized implementation study performed in real-world settings is that it uses a matched comparison group to enhance the evaluation of the AICP outcomes. Another strength is that the study uses the RE-AIM framework for evaluating the results in terms of public health impacts. Also, the use of qualitative methods to ascertain the perceived feasibility and satisfaction regarding AICP implementation from the perspective of professionals and participants will help us to understand why this pathway works (or does not work) and identify the essential components or strategies that require optimization. On the other hand, the relatively small number of health centres that are to receive the intervention, from only two HDs not fully representative of all centres in our regional health service, is a relevant limitation as it has implications for the generalization of our findings. Nonetheless, it allows standardization of the AICP implementation, thereby increasing the study's internal validity. In the case of successful results, health planners, managers, health professionals and citizens will have valid scientific evidence that justifies the incorporation of the fundamental methodological innovations in the emerging field of Implementation Science, as a way to facilitate the adoption and implementation of Clinical Care Pathways. The results obtained will inform the subsequent scaling up to all Osakidetza in order to increase the scope of good practices and their health outcomes of the users of our paediatric services.

## 4 Ethics and dissemination

This research protocol was reviewed and approved by the Basque Country Clinical Research Ethics Committee (Reference: PI2019121, approved on 23 October 2019). The study will be conducted according to the Declaration of Helsinki guidelines, to International Guidelines and to current Clinical Trial laws. The study protocol was registered in ClinicalTrials.gov (ID: NCT06437444, registered 7 May 2024, Last update 3 March 2025).

**Figure 2:**
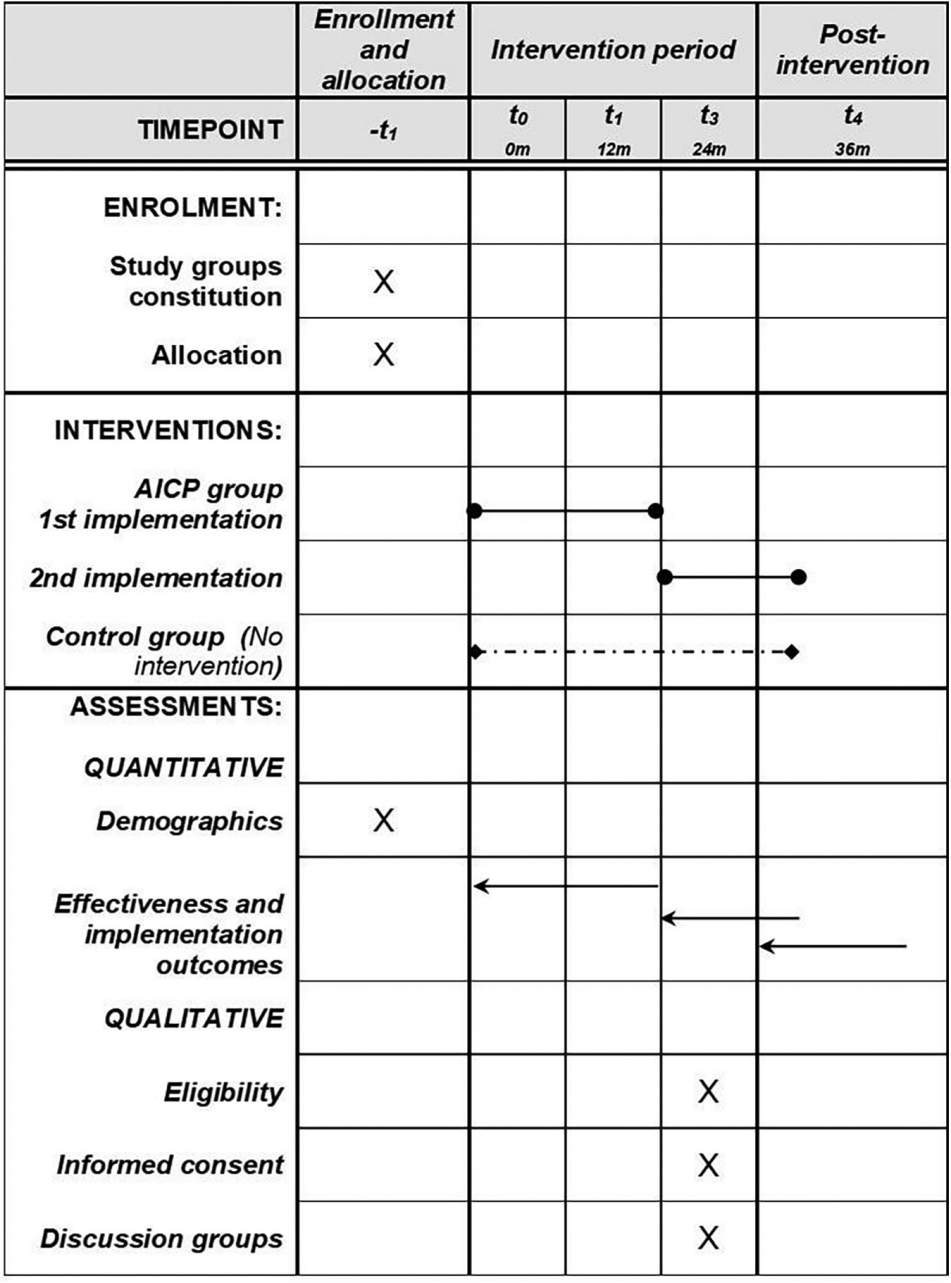
SPIRIT schedule of procedures. AICP, asthma integrated care pathway.

We used the Standard Protocol Items: Recommendations for Interventional Trials (SPIRIT) reporting guidelines and the SPIRIT checklist when writing this protocol (39). (see Figure 2; Supplementary File S7)

Since data supporting the present study will mostly concern routine data retrieved from EHR of Osakidetza, it will be only shared on justified request to the study guarantors. The results of the study will be published in indexed scientific journals, regardless of whether they are positive, negative, or inconclusive at the end of the study.
