# Supplementary material for: Improving the management of acute asthma in children through an integrated care pathway: an implementation study protocol
Source: Front Pediatr. 2025 Aug 19;13:1646499. doi: 10.3389/fped.2025.1646499 (PMC12403179; doi:10.3389/fped.2025.1646499)
Supplement: Supplementary file 1 [file Supplementaryfile1.docx]

**Supplementary Table 1a.** Actions carried out from May 7 2023 to May 6 2025

| **Interventions/Actions** | **Description** |
| --- | --- |
| **Contents of the care pathway** | - WHY: To inform and disseminate to paediatric professionals (both physicians and nurses) up-to-date evidence, which is the basis of current clinical practice guidelines, on the management of asthma exacerbations - WHAT: - *Materials*: summary document (PowerPoint) with all the recommendations proposed by the care pathway (including the literature references on which the pathway itself is based), together with an algorithm summarising the decision-making process to be followed - *Procedures*: dissemination to paediatric professionals of both the decision-making algorithm and the document containing all the clinical recommendations to be followed for the management of paediatric asthma exacerbations - WHO PROVIDED: The group of paediatric professionals promoting and coordinating the care pathway - HOW: Online. Individual mailing to each paediatric professional - WHERE: Transmitted via corporate mail - WHEN and HOW MUCH: - *WHEN*: from 7 May 2023 - *HOW MUCH*: material is sent once |
| **Computer tool** | - WHY: To remind paediatric professionals to perform and record the procedures recommended by the care pathway - WHAT: - *Materials*: Computer software - *Procedures*: After selecting the diagnosis of ‘Asthma with exacerbation (Acute)’ in the clinical history management software (Osabide Global), a pop-up window appears showing the actions to be taken as recommended by the care pathway. These recommendations include recording the administration of bronchodilators with metered-dose inhalers, assessing and recording the severity of each episode, assessing and recording persistent asthma symptoms, initiating background treatment when such symptoms are present, and training patients and their families in the inhalation technique and management of the disease - WHO PROVIDED: The health service's computer services - HOW: Online. Notification tool added to the health service's medical record management programme (Osabide Global) - WHERE: In the health record management software (Osabide Global) - WHEN and HOW MUCH: - *WHEN*: from 7 May 2023 - *HOW MUCH*: each time a paediatrician records a diagnosis of ‘Asthma with exacerbation (Acute)’ |
| **Patient educational material** | - WHY: to train and educate patients and their families in the management of asthma exacerbations, including information about the disease and the inhalation technique needed for the treatment of asthma - WHAT: - *Materials*: infographics and documents with information - *Procedures*: After selecting the diagnosis of ‘Asthma with exacerbation (Acute)’ in the patient electronic health record management tool, a reminder appears showing the documents to be given to families - WHO PROVIDED: paediatric professionals - HOW: Face-to-face, during patients' visits to the health service - WHERE: In the software tool - WHEN and HOW MUCH: - *WHEN*: from 7 May 2023 - *HOW MUCH*: it depends on the number of visits by each patient |
| **Training courses (paediatrics)** | - WHY: To raise awareness of the key aspects of the care pathway, including the initial assessment, treatment and follow-up of paediatric patients with acute asthma - WHAT: - *Materials*: Presentation (PowerPoint), paper questionnaire - *Procedures*: Oral presentation of the care pathway and key aspects of care, including the appropriate approach to patients (PAT and ABCDE sequence), assessment of the severity of each episode, treatment, inhalation technique, assessment and recording of persistent symptoms, patient follow-up, and patient/family education. Presentation of clinical cases. Final test questionnaire. - WHO PROVIDED: The group of paediatric professionals promoting and coordinating the care pathway - HOW: Online group courses (maximum 30 places) - WHERE: Hospital San Eloy, in a room with projection equipment - WHEN and HOW MUCH: - *WHEN*: between 15 May 2023 and 28 May 2023, and between 27 March 2025 and 1 April 2025 - *HOW MUCH*: 7.5 hours |
| **Training courses (nursing)** | - WHY: To raise awareness of the key aspects of the care pathway, including the initial assessment, treatment and follow-up of paediatric patients with acute asthma - WHAT: - *Materials*: Presentation (PowerPoint), paper questionnaire - *Procedures*: Oral presentation of the care pathway and key aspects of care, including the appropriate approach to patients (PAT and ABCDE sequence), treatment, inhalation technique, chamber types and maintenance, patient follow-up, and patient/family education; presentation of clinical cases; and final test questionnaire - WHO PROVIDED: The group of paediatric professionals promoting and coordinating the care pathway - HOW: Online group courses (maximum 30 places) - WHERE: Hospital San Eloy, in a room with projection equipment - WHEN and HOW MUCH: - *WHEN*: between 15 May 2023 and 24 May 2023, and between 8 April 2025 and 9 April 2025 - *HOW MUCH*: 4.5 hours |
| **Audit & Feedback reports** | - WHY: To get health professionals to change their care practice by adhering to the recommendations of clinical practice guidelines and the care pathway itself - WHAT: - *Materials*: reports with content including prescribing rates and recording of indicators highlighted by care pathway - *Procedures*: circulation by corporate email - WHO PROVIDED: The referents (professionals involved with the pathway) of each health centre - HOW: Circulation by corporate email - WHERE: Through corporate email - WHEN and HOW MUCH: - *WHEN*: between 7 May 2023 and 6 May 2025 - *HOW MUCH*: monthly |
| **Newsletter (email circular)** | - WHY: To raise awareness of the care pathway and its initiatives, with the aim of getting healthcare professionals to change their care practice by adhering to the recommendations of the clinical practice guidelines and the care pathway itself - WHAT: - *Materials*: notifications, messages, reminders and infographics with care pathway messages - *Procedures*: sending of both reminders and messages related to the care pathway, as well as material produced by the care pathway - WHO PROVIDED: The group of paediatric professionals promoting and coordinating the care pathway - HOW: Circulation by corporate email - WHERE: Through corporate email - WHEN and HOW MUCH: - *WHEN*: between 7 May 2023 and 6 May 2025 - *HOW MUCH*: Two mailings |

HD: health district; PAT: Pediatric Assessment Triangle

**Supplementary Table 1b.** Actions carried out from May 7 2024 to May 6 2025

| **Interventions/Actions** | **Description** |
| --- | --- |
| **Patient educational material** | - WHY: to train and educate patients and their families in the management of asthma exacerbations, including information about the disease and the inhalation technique needed for the treatment of asthma - WHAT: - *Materials*: videos, infographics, and documents with information - *Procedures*: After selecting the diagnosis of ‘Asthma with exacerbation (Acute)’ in the patient electronic health record management tool, a reminder appears showing the documents to be given to families. In addition, on the posters placed in the consultation and waiting rooms of the health centres, families can scan a QR code to access this content - WHO PROVIDED: the paediatric professionals - HOW: Face-to-face, during patients' visits to the health service - WHERE: In the electronic health record management tool and via QR codes on the posters - WHEN and HOW MUCH: - *WHEN*: from 7 May 2024 - *HOW MUCH*: at each patient visit |
| **Training content uploaded to the Intranet** | - WHY: to make paediatric professionals aware of the existence of both the care pathway and the contents developed both for professionals themselves and for patients and their families - WHAT: - *Materials*: 15 videos, 7 infographics and 8 presentations with educational content on the care pathway and its recommendations - *Procedures*: each paediatric professional of the two exposed HDs was notified by email and through the ‘Asma Bat’ WhatsApp Community of the existence of this content - WHO PROVIDED: The information technology services of each HD in collaboration with the professionals in charge of designing the care pathway - HOW: from 7 May 2024, the contents will be available for the paediatric professionals of the two HDs exposed - WHERE: on the corporate intranet of the two HDs exposed to the care pathway - WHEN and HOW MUCH: - *WHEN*: between 7 May 2024 and 6 May 2025 - *HOW MUCH*: 15 videos, 7 infographics and 8 presentations with educational content on the care pathway and its recommendations |
| **Training pills (WhatsApp community)** | - WHY: To raise awareness of the care pathway and its initiatives, with the aim of getting healthcare professionals to change their care practice by adhering to the recommendations of the clinical practice guidelines and the care pathway itself - WHAT: - *Materials*: notifications, messages, reminders, videos and infographics with care pathway messages - *Procedures*: sending reminders, care pathway messages and care pathway material - WHO PROVIDED: The group of paediatric professionals promoting and coordinating the care pathway - HOW: Online. Circulation via the ‘Asma Bat’ WhatsApp community - WHERE: Through the ‘Asma Bat’ WhatsApp community - WHEN and HOW MUCH: - *WHEN*: between 7 May 2024 and 6 May 2025 - *HOW MUCH*: weekly dispatch |
| **Posters** | - WHY: to notify and remind of the existence of the care pathway and its recommendations - WHAT: - *Materials*: 6 posters - *Procedures*: The posters are placed in consultation and waiting rooms so that they can be seen by paediatricians, as well as patients and their families - WHO PROVIDED: paediatricians themselves are the ones who place them in their health centres - HOW: on-site - WHERE: In consultation and waiting rooms - WHEN and HOW MUCH: - *WHEN*: from 5 September 2024 to 6 May 2025, coinciding with the beginning of the peak of cases in our health service - *HOW MUCH*: 6 posters with the following messages:   *1*. ‘NEW PAEDIATRIC ASTHMA CARE PATHWAY: Sharing the same way of working makes us stronger’.  2. ‘WITHOUT DATA, WE ONLY HAVE AN OPINION’  3. ‘STAY UP-TO-DATE. New Paediatric Asthma Care Pathway. Get updates about the new care pathway on your mobile. Join the WhatsApp group.’  4. ‘NEW PAEDIATRIC ASTHMA CARE ROUTE. We work in the same way to maintain our commitment to the youngest children.’  5. ‘WE ARE YOUR BEST SOURCE OF INFORMATION ON PAEDIATRIC ASTHMA. Ask your professional for information about the new Paediatric Asthma Care Pathway.’  6. ‘BREATHE. YOU ARE IN THE BEST HANDS.’ |

HD: health district
