# Supplementary material for: Improving the management of acute asthma in children through an integrated care pathway: an implementation study protocol
Source: Front Pediatr. 2025 Aug 19;13:1646499. doi: 10.3389/fped.2025.1646499 (PMC12403179; doi:10.3389/fped.2025.1646499)
Supplement: Supplementary file 3 [file Supplementaryfile3.pdf]

AICP Logic Model

Initial conditions	Inputs		Proximal outcome		Distal outcomes
**Problem**	**Initiative**	**Strategies**	**Mechanisms**	**Implementation outcomes**	**Clinical outcomes**
Care provided to children presenting acute asthma episodes not in accordance with clinical practice recommendations and with high degree of variability among professionals and care settings	Asthma Integrated Care Pathway (AICP)	AICP workflow and supporting documents<br><br>Computerized support tools<br><br>Training courses for professionals<br><br>Regular audit/feedback<br><br>Multi-channel reminders (newsletter, training pills, posters, etc.)<br><br>Patient education material	Raising awareness among health care professionals and users<br><br>High-quality evidence-based knowledge and skills<br><br>Provider commitment and shared goals<br><br>Families empowerment<br><br>Other facilitators and barriers at the intervention, user, professional, or organizational level	Increased adoption and implementation by professionals and reach among users:<br><br>Assessment and recording of the Pulmonary Score;<br><br>Assessment and recording of asthma symptoms using the PACT;<br><br>Administration of background treatment in cases of persistent asthma symptoms;<br><br>Administration of bronchodilators using an MDI with a spacer chamber in children diagnosed with a mild-to-moderate asthma attack.	Improved care provided to children presenting acute asthma episodes and reduced the variability among professionals and care settings in accordance with clinical practice guideline recommendations
