# Supplementary material for: Improving the management of acute asthma in children through an integrated care pathway: an implementation study protocol
Source: Front Pediatr. 2025 Aug 19;13:1646499. doi: 10.3389/fped.2025.1646499 (PMC12403179; doi:10.3389/fped.2025.1646499)
Supplement: Supplementary file 4 [file Supplementaryfile4.pdf]

Guía de codificación de determinantes del comportamiento – Theoretical Domains Framework

Dominio	**Constructos relacionados**	**Descripción**
Conocimiento (K)	Conocimiento<br>Conocimiento sobre el resultado / justificación científica<br>Esquemas y mentalidades y representaciones de enfermedades<br>Conocimiento procesal	Expresa explícitamente que tienen conocimiento, conocimiento o familiaridad (o ninguno) sobre el documento guía, evidencia o comportamiento objetivo.<br>Expresión de confianza o confianza en el documento guía, evidencia o comportamiento objetivo
Habilidades (S)	Habilidades<br>Competencia / habilidad<br>Desarrollo de habilidades / práctica<br>Habilidades interpersonales<br>Estrategias de afrontamiento<br>Evaluación de habilidades	Expresa explícitamente la capacidad de hacer algo / una habilidad observable.<br>Solo codifique aquí si es realmente observable y no es una percepción de una habilidad (lo que la convierte en una capacidad)
Creencias sobre capacidades (CA)	Autoeficacia/Competencia percibida/Control conductual percibido<br>Control de comportamiento<br>Control del medio ambiente (material y social)<br>Autoestima<br>Autoconfianza / confianza profesional<br>Optimismo / pesimismo<br>Empoderamiento	Percepción de facilidad / dificultad para llevar a cabo el comportamiento.<br>Percepciones sobre su propia competencia o la de otros y confianza para hacer un comportamiento.
Creencias sobre las consecuencias (CO)	Expectativas de resultados<br>Arrepentimiento anticipado<br>Optimismo poco realista<br>Valoración / evaluación / revisión<br>Actitudes/Creencias<br>Contingencias<br>Refuerzo / castigo / consecuencias<br>Incentivos / recompensas<br>Eventos destacados, incidentes críticos, sensibilización	Las características de las expectativas de resultados incluyen: física, social, emocional; proximal / distal, valorado / no valorado, probable / improbable, riesgo / recompensa; saliente / no saliente<br>Creencias, actitudes y expectativas con respecto a los resultados y las consecuencias del comportamiento (ya sea positivo o negativo) para uno mismo y para los demás.<br>Esto a veces puede confundirse con SI, debe ser claro si se trata de las consecuencias del comportamiento en el paciente / profesional (es decir, las consecuencias) o la influencia del comportamiento / actitudes de los demás en el profesional (es decir, la influencia social)<br>Cosas como una relación con el paciente se clasifica como SI, ya que facilita el comportamiento (no es una consecuencia del comportamiento en sí mismo y, por lo tanto, no se clasifica como CO).
Motivación, objetivos, intención (MI)	Intención: estabilidad / certeza<br>Objetivos (autónomos / controlados)<br>Establecimiento de objetivos<br>Metas proximales y distales<br>Motivación intrínseca<br>Compromiso<br>Etapas de cambio<br>Prioridad del objetivo	Deseo o deseo de llevar a cabo el comportamiento.<br>Priorizarlo por encima de otros comportamientos.<br>Intención de realizar el comportamiento.
Memoria, atención, toma de decisiones (MAD)	Memoria<br>Atención/Control de la atención<br>Toma de decisiones	Atención / recordar realizar un comportamiento

Contexto ambiental, recursos, limitaciones (E)	Recursos (materiales, temporales, espaciales, personal): disponibilidad<br>Administración de recursos<br>Estresores ambientales<br>Interacción persona x ambiente<br>Conocimiento del entorno de la tarea	Factores relacionados con la cirugía que influyen en si se está llevando a cabo o no un comportamiento (por ejemplo, disponibilidad de tiempo, espacio, personal, equipo, etc.)<br>Si hay una gran carga de caries, esto se codifica aquí, ya que esto tiene un impacto en el tiempo disponible para hacer la prevención.
Rol e identidad social y profesional (SPI)	Identidad/Identidad profesional / límites / rol<br>Identidad grupal / social<br>Normas sociales / grupales<br>Alienación<br>Compromiso organizacional	Identidad profesional (rol, límites de responsabilidades, obligaciones)<br>Percepción del rol propio dentro del contexto de la práctica / equipo dental (por ejemplo, enfermeras dentales que se sienten sin poder)<br>El punto con esto es que son cosas intrínsecas a la persona, su percepción de sí mismas como un rol profesional particular.
Influencias sociales (SI)	Apoyo social: interpersonal, profesional, organizacional, sociedad / comunidad<br>Normas sociales / grupales<br>Clima organizacional, cultura.<br>Gestión, gestión del cambio. Administración de recursos<br>Liderazgo<br>Trabajo en equipo, conflicto, demandas / roles competitivos<br>Supervisión, retroalimentación<br>Aprendizaje y modelado<br>Campeones, modelos a seguir<br>Límites profesionales, roles<br>Conflicto entre grupos, comparaciones sociales Identidad grupal / social, conformidad, alienación<br>Negociación<br>Presión social, poder, jerarquía	Opiniones y comportamiento de personas ajenas a la práctica (por ejemplo, pacientes) que altera la forma en que se realiza (o no se realiza) un comportamiento.<br>Otras personas que esperan o no esperan el comportamiento.<br>Otras personas que influyen (facilitan o inhiben) el comportamiento<br>Observación del comportamiento en otros que influyen en el propio comportamiento.<br>A diferencia de SPI, este dominio incluye cosas externas a la persona, no sus percepciones de su rol, sino las actitudes y el comportamiento de otras personas que tienen un efecto en la capacidad de la persona para llevar a cabo el comportamiento.<br>Hasta ahora, la codificación ha identificado la influencia social principalmente en términos de la influencia de los pacientes en lugar de la gestión, etc.
Emoción (em)	Afecto: positivo, negativo<br>Estrés<br>Arrepentimiento anticipado<br>Miedo<br>Sobrecarga, fatiga, agotamiento<br>Amenaza percibida, Ansiedad, depresión	Beneficios / riesgos emocionales al hacer un comportamiento, tales como indiferencia, no hay recompensa por hacer un comportamiento
Regulación de comportamiento (BR)	Objetivo, establecimiento de objetivos. Planificación de la acción<br>Intención de implementación<br>Moderadores de la brecha intención-comportamiento<br>Generando alternativas<br>Autocontrol<br>Priorizando<br>Retroalimentación<br>Barreras y facilitadores	Cosas que las personas hacen para ayudarse a sí mismas a realizar el comportamiento / facilitar el comportamiento<br>Rutinas y sistemas (formales o informales) asociados con el desempeño o la modificación de un comportamiento.<br>Formas de trabajo que hacen que sea más fácil llevar a cabo el comportamiento previsto a nivel individual u organizacional.<br>Estrategias para facilitar y apoyar el comportamiento, modificar un comportamiento existente o introducir un nuevo comportamiento
Naturaleza del comportamiento (NB)	Rutina, automática, hábito<br>Rompiendo un hábito<br>Experiencia directa, comportamiento pasado	Una descripción de lo que la persona generalmente hace en la actualidad
