# Supplementary material for: Improving the management of acute asthma in children through an integrated care pathway: an implementation study protocol
Source: Front Pediatr. 2025 Aug 19;13:1646499. doi: 10.3389/fped.2025.1646499 (PMC12403179; doi:10.3389/fped.2025.1646499)
Supplement: Supplementary file 6 [file Supplementaryfile6.docx]

***Hoja de Información al Paciente y Consentimiento Informado***

**Título:** Crisis asmática en pediatría: Impacto de una ruta asistencial en atención primaria y hospitalaria

**Investigador Principal:** Marta Montejo Fernández

**Servicio/Centro:** Subdirección para la coordinación de atención primaria/Unidad de investigación atención primaria-IIS Biobizkaia

**Entidad financiadora:** Departamento de Salud Gobierno Vasco/ Instituto de salud Carlos III

Apreciado Sr./a,

Osakidetza-Servicio Vasco de Salud, con el propósito de mejorar la calidad en la prestación de servicios de salud hacia la ciudadanía, le invita a participar en el estudio “Crisis asmática en pediatría: Impacto de una ruta asistencial en atención primaria y hospitalaria”.

Antes de decidir si desea participar, es importante que entienda los objetivos, la importancia de su participación y en qué consistirá, además de qué uso se dará a los datos recogidos y los posibles beneficios y riesgos.

Léalo atentamente y consulte cualquier duda con los miembros del equipo de investigación.

1. **OBJETO DEL GRUPO DE DISCUSIÓN**

El objetivo de los grupos de discusión del Proyecto de la Ruta Asistencial Integrada del Asma es generar conocimiento –a través de las percepciones de los/las pacientes de pediatría sobre la práctica clínica en las crisis asmáticas. A través de una serie de preguntas abiertas se analizarán diferentes aspectos relacionados con la experiencia percibida por los/las pacientes con la atención recibida, tratando de conocer la opinión de todos los integrantes del grupo sobre este tema.

No existen respuestas buenas o malas. Cualquier integrante del grupo está invitado a expresar libremente su opinión y a respetar la de los otros integrantes, aunque sea diferente de la suya.

1. **PARTICIPACIÓN Y RETIRADA DEL ESTUDIO**

Este estudio está aprobado por el Comité de Ética de la Investigación con Medicamentos de Euskadi (CEIm-E). Su participación en el mismo es voluntaria y en cualquier momento puede decidir abandonarlo, aunque haya proporcionado el consentimiento y el estudio esté en pleno desarrollo. Su decisión no afectará la atención sanitaria que reciba posteriormente. Además, usted tiene derecho a solicitar al equipo investigador del estudio, en cualquier momento, y sin necesidad de especificar el motivo, la eliminación de sus datos. Su participación en este estudio no supondrá para usted ningún coste económico, así como tampoco será recompensado económicamente por ello.

1. **DESARROLLO DEL ESTUDIO**

Se realizará una sola entrevista llevada a cabo por dos investigadores con experiencia en métodos de investigación cualitativa, así como en el campo clínico y el proyecto. En dicha entrevista se le harán preguntas sobre su experiencia y satisfacción con el servicio recibido en crisis asmáticas.

La discusión grupal será grabada (en formato audio) con el fin de transcribirla íntegramente. Esto permite a los miembros del equipo participar en la discusión sin necesidad de tomar notas, evitándose así el riesgo de no reflejar fidedignamente las opiniones expresada por los miembros del grupo.

1. **USO Y CONFIDENCIALIDAD DE LOS DATOS**

Los datos que se obtengan en el grupo de discusión se utilizarán únicamente con fines de investigación y solamente por parte del equipo de investigación de la Unidad de Investigación de Atención Primaria de Bizkaia (UIAPB). Todas las opiniones expresadas por los/las participantes serán tratadas de manera anónima y confidencial. Se le informa de que no se va a recoger ningún dato de carácter personal.

El estudio cumple lo establecido en el REGLAMENTO (UE) 2016/679 DEL PARLAMENTO EUROPEO Y DEL CONSEJO de 27 de abril de 2016 relativo a la protección de las personas físicas en lo que respecta al tratamiento de datos personales y a la libre circulación de estos datos. Se le solicita también su consentimiento para la realización de este proyecto de investigación conforme a las exigencias del Reglamento Europeo 2016/679 de Protección de Datos y a la Ley Orgánica 3/2018, de 5 de diciembre, de Protección de Datos Personales y garantía de los derechos digitales que deroga la Ley Orgánica 15/1999, de 5 de diciembre, de protección de datos personales. No se cederán datos a terceros, salvo obligación legal.

Si usted tiene alguna duda o requiere cualquier tipo de información no dude en contactar con

el/la médico que le informa, Dr./a ___________________________________, cuyo lugar de trabajo

es el Servicio de______________________________ del Hospital Universitario

_______________; teléfono: __________ (extensión___________).

Usted también puede contactar con el Investigador Principal responsable:

**Nombre:** Marta Montejo Fernández

**Teléfono:** 946006671

**e-mail:** [marta.montejofernandez@osakidetza.eus](mailto:marta.montejofernandez@osakidetza.eus)

**Dirección:** Edificio Biocruces 3, Plaza Cruces 12, 48903

1. **DECLARACION DEL CONSENTIMIENTO INFORMADO**

**Título:** Crisis asmática en pediatría: Impacto de una ruta asistencial en atención primaria y hospitalaria

**Investigador Principal:** Marta Montejo Fernández

Investigador/a médico/a:…………………………………………………..

**Servicio/Centro:** Subdirección para la coordinación de atención primaria/Unidad de investigación atención primaria-IIS Biobizkaia

Yo, Don/Doña…………………………………………………….(nombre y apellidos del paciente), he leído este documento, he comprendido las explicaciones en él facilitadas acerca de la grabación del grupo de discusión y he podido resolver todas las preguntas que he planteado al respecto. Comprendo que mi participación en este ensayo es voluntaria y que puedo retirarme en cualquier momento.

También he sido informado/a de que mis datos personales serán protegidos y serán utilizados únicamente con fines de investigación por el equipo de investigadores de la Unidad de Investigación de Atención Primaria de Bizkaia (UIAPB).

Tomando todo ello en consideración y en tales condiciones, CONSIENTO participar en el grupo de discusión, en la grabación del mismo y en que los datos que se deriven de mi participación sean utilizados para cubrir los objetivos especificados en el documento.

**EN CONSECUENCIA, DOY MI CONSENTIMIENTO PARA PARTICIPAR EN ESTE PROYECTO DE INVESTIGACIÓN.**

………………………………………………….. ………………………………………………………..

Firma del/la paciente Firma del/la médico responsable

…………..………………....................... …………..………………............................

Nombre y apellidos Nombre y apellidos

Fecha ……/………/20………… Fecha ……/………/20…………
